# On Prurigo

**Published:** 1805-12

**Authors:** 


### *To the Editors of the Medical and Physical Journal.*

Gentlemen,

THE eruption which is occasionally seen upon children who have undergone vaccination, (though by no means peculiar to them) and which has lately been denominated the *cow-pock itch or mange*, is very exactly described by Dr. Willan, in his Classification of Cutaneous Diseases, Order I. under the name, *Prurigo.*

" The Prurigo originates without any previous indisposition, generally in spring or the beginning of summer. It is characterized by soft and smooth elevations upon the skin, which seldom appear red, or much inflamed, except from violent friction. They are accompanied with a sense of itching almost incessant; this is however felt more particularly on undressing, and often prevents rest for some hours after getting into bed. When the tops of the papulæ are removed by rubbing or scratching, a clear fluid oozes out from them, and gradually concretes into thin black scabs. Notwithstanding this change, the itching does not abate, and by the constant friction, which it demands, inflamed pustules are sometimes produced.

" This species of prurigo mostly affects young persons. Those who have originally a delicate or irritable skin, must in the same circumstances be the greatest sufferers.

" The

"The eruption extends to the arms, breast, back and thighs; and often continues during two or three months of the summer. When persons affected with it, neglect washing the skin, or are uncleanly in their apparel, the eruption grows more inveterate, and at length changing its form, often terminates in the itch. Pustules arise among the papulæ, some filled with lymph, others with pus; the acarus scabiei begins to breed in the furrows of the cuticle; and the disorder becomes contagious."*

The above described disease is well known to arise from a great variety of causes; I have never been able to trace it as evidently originating from vaccination, nor can I comprehend the arguments of those gentlemen who contend for the reality of this origin.

If for the sake of argument we allow the possibility that these eruptions may be the consequence of vaccination, I do not know how to get over the difficulty of accounting for their appearing so rarely; how happens it that only one child in a series of inoculation with the same virus should be thus attacked? A is inoculated with vaccine virus, passes through the disorder regularly, and has no eruption afterwards. B, C, D, and E, are inoculated from A; E only has an after-eruption; from E are inoculated F, G, H, and I, not one of whom has the least appearance of eruption. Can any one, then, in the habit of reasoning about the causes of diseases, contend, that the eruption upon E, was the consequence of vaccination? If the eruption be the *necessary* consequence of introducing vaccine virus into the system, such effect should (according to every known law of the propagation of disease) be constantly, or at least much more generally, produced.

Another difficulty to be solved, is the circumstance of the great difference in regard to the length of time after inoculation before the appearance of the eruption. Cutaneous diseases in some children have been attributed to the cow-pock, although generally several months, and in some instances, even years had elapsed after the inoculation, before any eruption was discoverable. How is this to be accounted for?

There can be no doubt, but that the learned author of "The rational and improved Practice of Physic," the exploder

* See the description and mode of Treatment at large in Dr. Willan's valuable work on Cutaneous Diseases, Order I. p. 71. This part was published in 1798; it is evident therefore, that the disease was well known long before the cow-pock had begun to be inoculated.

ploder of all false doctrines, has duly considered this subject; and can give the most cogent reasons in support of the opinion which he so publicly and peremptorily maintains; unfortunately they are not to be found in his late publication on the Cow-pock Mange, &c. May I be permitted to express a wish that this enlightened Physician would favour the Readers of your Journal with some Observations on the particulars above mentioned. An elucidation of these difficulties will be of much general utility as well as a favour conferred on

AN HUMBLE ENQUIRER

*November* 10, 1805.

